# Frailty Index Progression Around Hypertension Diagnosis in 14 Countries

**DOI:** 10.1161/HYPERTENSIONAHA.126.27590

**Published:** 2026-09-30

**Authors:** Dongfeng Tang, Aicha Goubar, Camila Pantoja-Ruiz, Ajay Bhalla, Julie Whitney, Miao Cai, Wuquan Deng, Shangfeng Tang, Iain J. Marshall, Charles D.A. Wolfe, Matthew D.L. O’Connell

**Affiliations:** Department of Primary Care & Data Science, School of Life Course and Population Sciences (D.T., A.G., C.P.-R., A.B., J.W., I.J.M., C.D.A.W., M.D.L.O.C.), King’s College London, United Kingdom.; Department of Ageing and Health, Guy’s and St Thomas NHS Foundation Trust (A.B.), King’s College London, United Kingdom.; Department of Clinical Gerontology, King’s College Hospital NHS Foundation Trust, London, United Kingdom (J.W.).; Clinical Epidemiology Center, Research and Development Service, VA Saint Louis Health Care System, Saint Louis, MO (M.C.).; Department of Endocrinology, School of Medicine, Chongqing University Central Hospital, Chongqing University, China (W.D.).; School of Medicine and Health Management, Huazhong University of Science and Technology, Hubei, China (S.T.).; Research Center for Rural Health Service, Key Research Institute of Humanities & Social Sciences of Hubei Provincial Department of Education, Hubei, China (S.T.).; NIHR Applied Research Collaboration (ARC) South London, United Kingdom (C.D.A.W.).

**Keywords:** aging, antihypertensive agents, frailty, hypertension, longitudinal studies

## Abstract

**BACKGROUND:**

Hypertension and frailty frequently coexist in older adults. However, relationships between their trajectories are unclear. This study aims to examine whether frailty progressed differently between individuals with and without hypertension before and after diagnosis.

**METHODS:**

Data were drawn from 3 nationally representative cohorts across 14 countries, including 21 418 participants (mean baseline age 63.2±10.2 years) followed for 16 to 18 years. Individuals with hypertension at baseline were excluded. Hypertension was defined by self-reported physician diagnosis and antihypertensive medication use by self-report of taking any medication. Diagnosis timing was based on the first survey wave at which hypertension was reported. Coarsened exact matching matched participants who developed hypertension with those who did not. Piecewise linear mixed models estimated frailty trajectories before and after diagnosis.

**RESULTS:**

Among 10 709 participants who developed hypertension (median diagnosis age 70 years), frailty index rescaled to a 0 to 100 scale (0–100) increased by 0.63 points/y (95% CI, 0.57–0.70) before diagnosis and 1.09 points/y (95% CI, 0.97–1.21) after diagnosis. During the 8 years preceding diagnosis, participants who developed hypertension experienced greater increases in frailty index rescaled to a 0 to 100 scale than matched controls without hypertension (difference, 2.69 [95% CI, 2.43–2.95]). The corresponding difference during the 8 years after diagnosis was 1.51 (95% CI, 1.01–2.01). Among individuals with hypertension, antihypertensive medication use was associated with a nonsignificant reduction in frailty progression after diagnosis. Findings remained robust after excluding participants with cardiovascular conditions.

**CONCLUSIONS:**

Participants with hypertension experienced faster frailty progression before and after diagnosis. Studies using objective blood pressure measurement are needed to confirm this association.

**Novelty and Relevance**

**What Is New?**

This study explored frailty trajectories both before and after clinical hypertension diagnosis across 3 harmonized aging cohorts spanning England, the United States, and European countries. Frailty accumulation was already accelerated in the 8 years preceding hypertension diagnosis, and this acceleration persists after diagnosis. Antihypertensive medication use was associated with a nonsignificant attenuation of postdiagnosis frailty progression.

**What Is Relevant?**

These findings shift the framing of the relationship between hypertension and frailty from a static, cross-sectional coexistence to a dynamic, time-dependent process. This study identified a clinically recognizable and potentially actionable period surrounding diagnosis for closer monitoring and early intervention.

**Clinical/Pathophysiological Implications?**

Frailty assessment could be incorporated into routine hypertension care at diagnosis and follow-up, enabling earlier identification of frailty and informing treatment decisions. Conversely, frail individuals should be considered for opportunistic blood pressure screening.

Frailty, defined as a multidimensional state of decreased physiological reserve and increased vulnerability, strongly predicts disability, hospitalization, and mortality in older adults.^1^ As a dynamic process rather than a fixed state, frailty reflects the cumulative impacts of biological aging, chronic disease, and environmental exposures over the life course.^2–4^

Hypertension is one of the most important risk factors for all-cause morbidity and mortality.^5^ Hypertension is often asymptomatic and may develop over many years, with a subclinical phase during which vascular and organ damage have already accumulated.^6–8^ Hypertension may drive progression of frailty, either through increased risk of cardiovascular morbidities, accumulation of hypertensive target organ damage, or shared mechanisms including arteriosclerosis, inflammation, and oxidative stress.^9–13^ Moreover, clinical diagnosis of hypertension often marks a turning point characterized by increased healthcare utilization, initiation of antihypertensive medication use, and behavioral change.^14^ These changes could possibly alter subsequent frailty trajectories, either mitigating functional decline through blood pressure (BP) control or accelerating the progression through treatment-related adverse effects and polypharmacy.^11,15^

Both hypertension and frailty increase in prevalence with age, and frequently coexist in older adults, with a meta-analysis estimating the prevalence of hypertension in frail adults to be 72%.^11,16^ Previous studies show that a population with hypertension was associated with higher prevalence or incidence of frailty.^16^ However, this association has not been consistently observed across studies.^16,17^ Emerging evidence suggests that poorly controlled BP, long-term elevated BP exposure, and greater BP variability may contribute to progression of frailty,^18–20^ while declining BP in older adults may also be associated with incidence of frailty.^21^ A more complex J-curve relationship between frailty and BP was observed in elderly outpatients with cardiometabolic diseases, and a decline in BP was associated with a slow walking speed and weak hand grip strength.^22^ In addition, frailty may modify the association between BP and clinical outcomes. Although hypertension combined with frailty has been linked to higher mortality in some studies,^23^ a systematic review did not consistently confirm this increased mortality risk.^24^ In contrast, elevated BP is associated with higher cardiovascular mortality in nonfrail older adults, whereas a potential protective effect of higher BP was found in frail populations.^25,26^ At last, limited studies explored the relationship between antihypertensive treatment and frailty, and results are inconclusive.^27^ Wu et al^28^ found greater odds for frailty incidence for those using calcium channel blockers, ACE (angiotensin-converting enzyme) inhibitors, angiotensin receptor blockers, diuretics, and β-blockers, but treatment with Aldosterone Antagonists was not associated with frailty incidence among elderly patients with hypertension. Meanwhile, Gray et al^29^ found no differences in incidence of frailty between current angiotensin-converting enzyme inhibitor users and nonusers among women aged ≥65 years.

Despite evidence above linking hypertension and frailty, the longitudinal association between hypertension and progression of frailty remains unclear. Most existing studies have examined BP as either a baseline or longitudinal exposure, including hypertension status, cumulative levels, variability, or predefined trajectories. However, fewer studies have explicitly anchored analyses around the clinical diagnosis of hypertension to examine how frailty trajectories evolve before and after diagnosis, which is a potential critical turning point. Furthermore, whether antihypertensive treatment modifies postdiagnosis progression of frailty is still unclear.

This study seeks to inform clinical strategies for the joint assessment and management of frailty and hypertension, to delay progression of frailty among hypertensive individuals. To address these gaps, this study aims to examine whether the progression of frailty differs between individuals with and without hypertension, both before and after clinical diagnosis, and whether antihypertensive medication use attenuates frailty progression after diagnosis of hypertension.

## Methods

### Data Availability

All data are publicly available and can be accessed at the following websites: the HRS (Health and Retirement Study; https://hrs.isr.umich.edu/), the SHARE (Survey of Health, Ageing and Retirement in Europe; https://share-eric.eu/), and the ELSA (English Longitudinal Study of Ageing; https://www.elsa-project.ac.uk/).

### Participants

Three aging cohort studies were used in this study: the US HRS (9 waves from wave 8 to16, 2006–2022), the ELSA (9 waves from wave 1 to 9, 2002–2018), and the SHARE (8 waves from wave 1–2, wave 4–9, 2004–2022). These HRS-family cohorts share comparable research protocols and nationally representative designs, including community-dwelling individuals aged ≥50 years and their partners (of any age). Detailed methods for these studies are published elsewhere.^30–32^

To match the length of follow-up, wave 1 of ELSA and SHARE, and wave 8 of HRS were set as the baseline surveys. Fourteen countries were included in the analysis: England (ELSA), United States (HRS), Austria, Germany, Sweden, the Netherlands, Spain, Italy, France, Denmark, Greece, Switzerland, Belgium, and Israel (all SHARE). Participants without hypertension at baseline, with at least 1 follow-up, were included in the analysis. Participants aged 50 years and over and younger partners aged at least 40 were included in the analysis, as hypertension onset is common between 40 and 50. A matched analysis sample with balanced characteristics across hypertension status was used. Other exclusion criteria were: missing hypertension data in any wave, as complete information was required to accurately determine hypertension status and the timing of diagnosis; missing covariate data at baseline; having >20% missing frailty index (FI) items. After exclusions, 21 418 participants providing 118 741 data points were included in the analysis (Figure S1).

### Measurement

Based on previous studies,^33,34^ frailty was measured using a 27-item FI based on the deficit accumulation approach. Different dimensions of individuals’ well-being, including self-reported health conditions, functional limitations, self-reported health status, and cognitive status, were used to construct the FI. Hypertension, stroke, or heart problems were not included in the FI for this analysis. Table S1 presents the full list of FI items. The FI was computed as the ratio of the number of present health deficits to the total number of items measured, yielding a continuous score from total fitness (0) to total frailty (1). FI was subsequently rescaled to a 0 to 100 scale (FI_100_) for modeling purposes.

In each wave, participants were asked to report whether they had been diagnosed with hypertension by a doctor since the last wave. Hypertension status was accordingly grouped as yes or no. The first year reporting hypertension was assumed to be the time point of diagnosis. Among participants with hypertension, respondents were additionally asked if they were taking medication for high BP at each wave. Antihypertensive medication status (yes/no) was accordingly defined among individuals, and participants with hypertension were classified as receiving antihypertensive medication if they reported current use of medication for high BP at any wave. Duration of untreated hypertension was additionally defined as the time interval between the first recorded hypertension diagnosis and the first recorded use of antihypertensive medication among individuals with hypertension postdiagnosis. Participants who were already receiving antihypertensive medication at the time of diagnosis were assigned a duration of 0 years. For participants who never reported antihypertensive medication use during follow-up, the duration of untreated hypertension was defined as the total postdiagnosis follow-up time.

Other covariates included age, sex (male, female), marital status (partnered, single), education (primary, secondary, and tertiary), household wealth quartiles, smoking (yes, no), drinking (yes, no), physical inactivity (yes, no), birth cohort (centered at the cohort born in 1940–1944), and country (categorical, indicating which country the participant came from). More details about the measurements of different variables are presented in the Supplemental Methods.

### Matching

To balance baseline characteristics across hypertension status, coarsened exact matching (CEM) was used to select the control group (no hypertension) based on age, marital status, sex, education, birth cohort, country, and FI at baseline, based on previous studies.^35^ This method has the advantages of being robust to measurement error, with fewer assumptions, and no need for an iterative checking process to guarantee the balance necessary for other methods.^35^ Participants diagnosed with hypertension were assigned to a matching stratum with those without hypertension based on the matching characteristics above. Then, participants were randomly dropped, and 2 groups (with or without hypertension) with an equal number of participants were created. The balance of different characteristics before and after matching was assessed using standardized mean differences (shown as %bias). More details about the matching process can be found in the Supplemental Methods.

### Statistical Analysis

Piecewise linear mixed effects models were used to model the differences in the progression of frailty before and after the diagnosis of hypertension. Mixed effects models use all available data regardless of length of follow-up, and handle nonmonotone missingness patterns and attrition, assuming data missing-at-random.^36^

Time was centered at the age of diagnosis (t=0) for the hypertension group, and the median age of diagnosis in the participant’s matching stratum for the no-hypertension group. Two time variables were defined to represent periods before (pretime, *t*<0) and after (posttime, *t*≥0) diagnosis. This specification allowed the rate of FI_100_ change to differ across the 2 periods within a single modeling framework. The main model included fixed effects for pretime, posttime, hypertension status, interaction of pretime and hypertension, and interaction of posttime and hypertension, enabling estimation of differences in the rate of change in FI_100_ between individuals with and without hypertension, both before and after diagnosis. The main model was also adjusted for age_*t*=0_ (centered at age 70 years, the median age of diagnosis in the hypertension group), birth cohort, sex, marital status, education, household wealth, country, smoking, drinking, and physical inactivity, along with interactions between these covariates and pretime and posttime (accounting for their effects on frailty progression before and after diagnosis). Random intercepts and random slopes for pretime and posttime were specified at the individual level, with an unstructured covariance matrix to account for heterogeneity in progression of frailty and correlations among random effects.

The slope of frailty trajectory among participants with hypertension before diagnosis was estimated from the pretime coefficient and the interaction of hypertension by pretime term. Similarly, the frailty slope after diagnosis was estimated from the coefficient of posttime and the interaction of hypertension by posttime term. The difference between frailty slopes before and after diagnosis among participants with hypertension was tested by calculating a linear contrast. Trajectories of frailty for participants with and without hypertension 8 years before and 8 years after diagnosis of hypertension were plotted using marginal means of FI_100_ and Stata’s margins command. In addition, to highlight the variation in frailty progression, differences in FI_100_ across hypertension status were calculated over the same 16-year window before and after diagnosis. To assess whether the difference in frailty slopes between individuals with and without hypertension changed after diagnosis, the difference-in-difference approach was used. This was implemented by subtracting the between-group difference in change of FI_100_ over the 8 years before diagnosis (defined as FI_100_ at t_0_ minus FI_100_ at t_0−8_) from the corresponding between-group difference over the 8 years after diagnosis (FI_100_ at t_0+8_ minus FI_100_ at t_0_), where t_0_ denotes the time of hypertension diagnosis.

To examine the effect of antihypertensive medication on frailty progression, another mixed-effects model was fitted among participants with hypertension. The model included fixed effects for posttime, antihypertensive medication status, and the interaction of posttime and antihypertensive medication status, enabling estimation of differences in rate of change in FI_100_ between individuals with and without taking antihypertensive medication after diagnosis. The main model was also adjusted for duration of untreated hypertension, age_t=0_, birth cohort, sex, marital status, education, household wealth, country, smoking, drinking, and physical inactivity, along with interactions between these covariates and posttime. Random intercepts and random slopes for posttime were specified at the individual level, with an unstructured covariance matrix. Trajectories of frailty for participants with and without medication use 8 years after hypertension diagnosis were plotted.

To explore the effect of age of diagnosis on the difference in frailty progression across hypertension status, an additional analysis was conducted by adding interaction between hypertension status, pretime and age_t=0_, and interaction between hypertension status, posttime and age_t=0_ to the main model. To assess which dimensions of frailty contributed to the observed associations of hypertension with FI, the 27 FI items were grouped into 4 domains: chronic conditions and physical health; activities of daily living and instrumental activities of daily living disabilities; mobility; and mental health and cognition. Detailed items included in different domains are presented in Table S3. A leave-one-domain-out sensitivity analysis was performed. For each of the 4 domains, the FI was reconstructed with all items in that domain excluded, and the primary analysis was repeated using the reconstructed FI. Estimated differences in the slope of frailty before hypertension diagnosis and after diagnosis were compared with those from the primary analysis to assess whether the observed associations were attributable to any individual frailty domain. To test the robustness of the results, some sensitivity analyses were conducted. First, analyses were repeated using the full sample with both matched and unmatched participants (N=29 886). Second, due to the potential for survival selection bias, analyses were repeated among participants with at least 3 follow-ups (n=11 418) and the subpopulation after excluding those with an FI≥0.25 (n=18 338), separately. Third, the analysis was replicated excluding participants with a history of stroke or heart problems at baseline and follow-ups (including angina, heart attack, congestive heart failure and other heart conditions) to avoid confounding effects of these cardiovascular events on frailty progression (n=14 308). Fourth, to determine whether the effect of antihypertensive medication remains robust among frail participants with hypertension, the analysis with antihypertensive medication and its interaction with posttime was repeated among participants in frail status (FI≥0.25) at baseline. Fifth, to address potential residual confounding by socioeconomic status, a separate CEM was performed in which household wealth quartiles were added to the original set of matching variables. The primary analysis was then reestimated.

## Results

The standardized mean differences of baseline characteristics between the hypertension and nonhypertension groups before and after CEM matching are shown in Table 1. Before matching, several covariates showed imbalances between groups, particularly household wealth, drinking status, and education. After CEM, the differences in baseline characteristics between the hypertension and control groups were substantially reduced. Standardized mean differences of all variables except household wealth were below 5%.

**Table 1.**
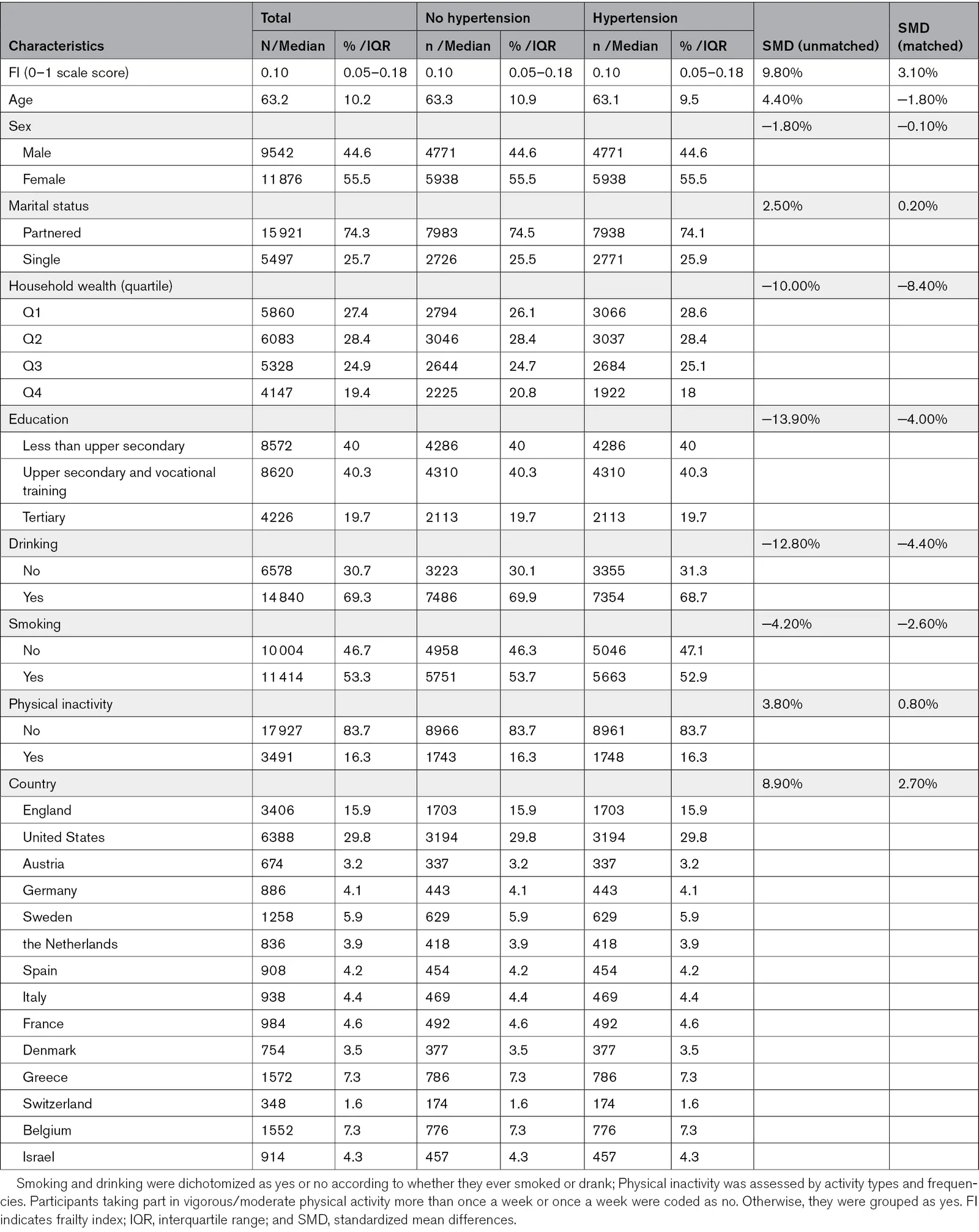
Baseline Characteristics of Participants

Table 1 also presents characteristics of participants. In total, 21 418 participants with a mean age of 63.2 (SD, 10.2) and a median FI score (0–1 scale) of 0.10 (interquartile range, 0.05–0.18) at baseline were included, with half of them eventually diagnosed with hypertension (10 709, 50.0%). Of the 14 countries included, the largest proportion of participants was from the United States (n=6388, 29.8%) and the smallest from Switzerland (n=348, 1.6%). Overall, 55.5% (n=11 876) of participants were female, 74.3% (n=15 921) were in a partnered status, and 19.7% (n=4226) had a tertiary education level at baseline. The characteristics of the analysis sample and participants excluded due to baseline hypertension are presented in Table S2. Excluded participants were older (mean age: 67.0 versus 63.1) and had higher FI (median FI: 0.15 versus 0.10, 0–1 scale).

Figure 1A presents frailty trajectories across hypertension status before and after diagnosis, and Figure 1B shows the corresponding differences in FI_100_ across hypertension status at different time points. Figure 1C shows that the averaged FI_100_ increases over age overall and across hypertension status. The average marginal change in FI_100_ was 0.63 (0.57–0.70) per year for participants with hypertension, and 0.29 (0.23–0.36) for participants without hypertension before diagnosis (Table 2). After the diagnosis of hypertension (t>0), the corresponding average marginal change was 1.09 (0.97–1.21) per year in the hypertension group, and 0.90 (0.77–1.03) in the no hypertension group (Table 2).

**Table 2:**
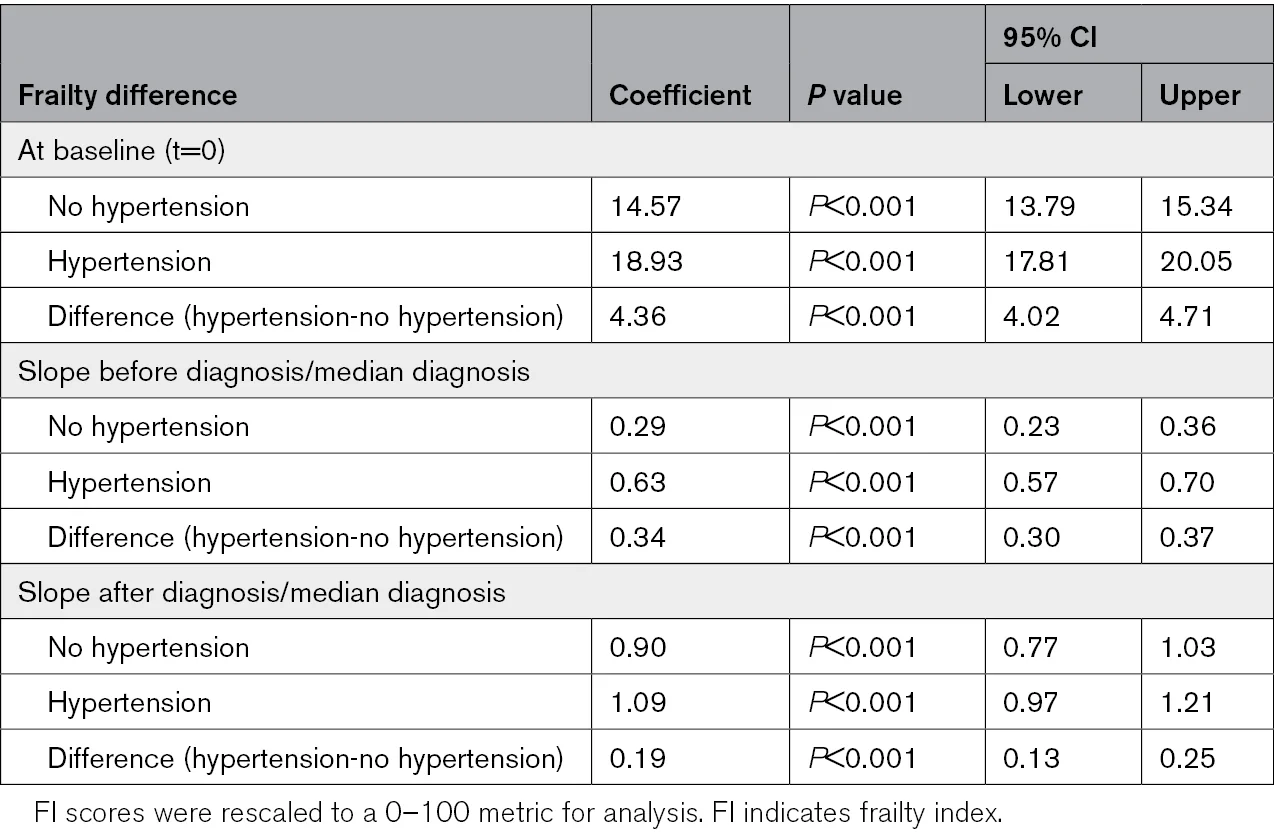
Mixed Effects Models for the Relationship Between Hypertension and Frailty Accumulation Over Time

**Figure 1.**
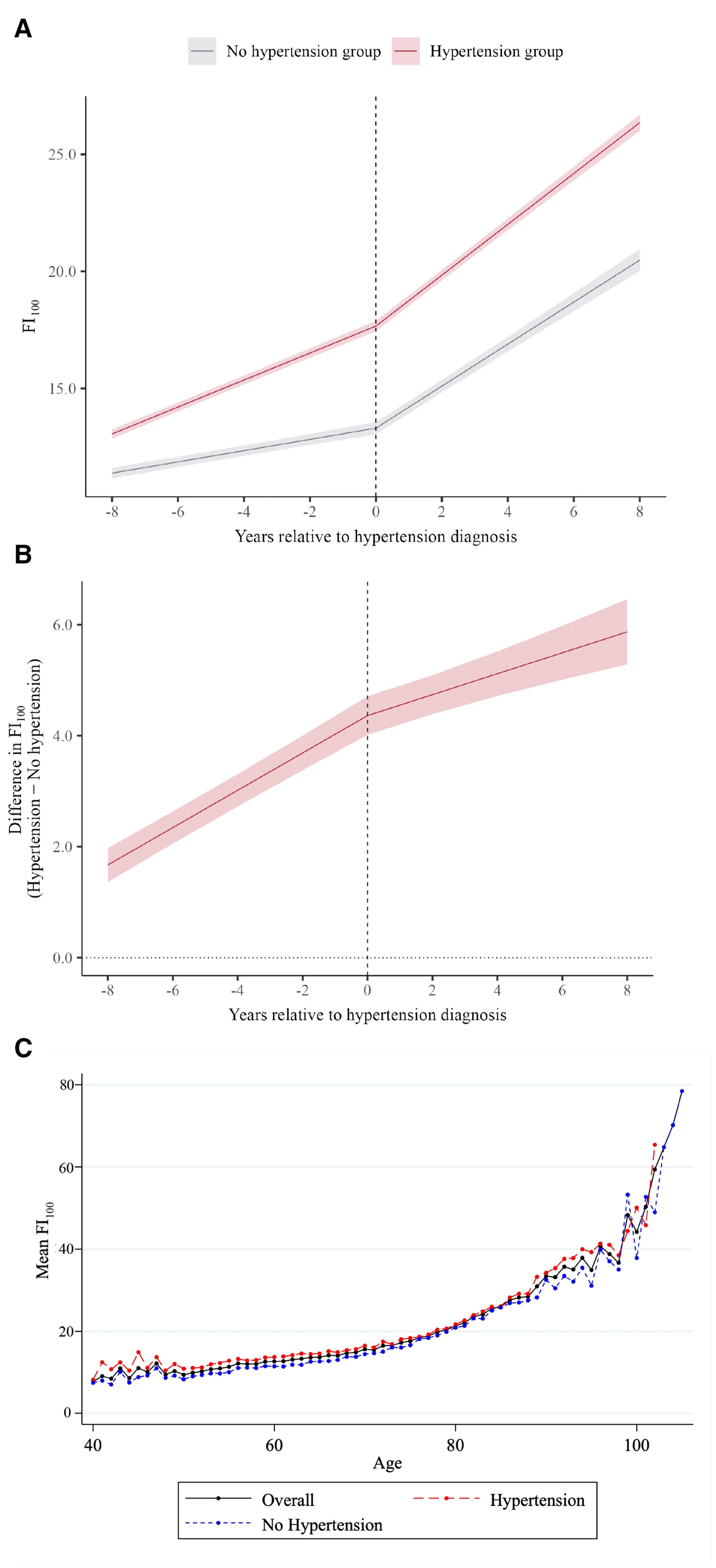
**FI rescaled to a 0 to 100 scale (FI)_100_ predicted by years relative to diagnosis of hypertension, differences in FI_100_ across hypertension status, and averaged FI_100_ increases over age. A**, Frailty trajectories around hypertension diagnosis. **B**, Differences in FI_100_ across hypertension status. **C**, Averaged FI_100_ increases over age.

Figure 2 presents the changes in FI_100_ before and after diagnosis in the hypertension and no hypertension groups. Over 16 years, participants with hypertension showed an increase of 13.76 points (95% CI, 12.73–14.80) in FI_100_, including 5.04 (95% CI, 4.52–5.57) in the 8 years before diagnosis and 8.72 (95% CI, 7.74–9.70) in the 8 years after diagnosis. Compared with participants with hypertension, those without hypertension had smaller changes in FI_100_, with a difference of 4.20 (3.67–4.73) in the increase of FI_100_ over 16 years. The corresponding difference in the 8 years before and after diagnosis was 2.69 (2.43–2.95) and 1.51 (1.01–2.01), respectively. The between-group difference in FI_100_ accumulation over the same 8-year period was significantly greater before diagnosis than after diagnosis, yielding a difference-in-difference estimate of 1.18 FI_100_ points (−1.78 to −0.59; *P*<0.001).

**Figure 2.**
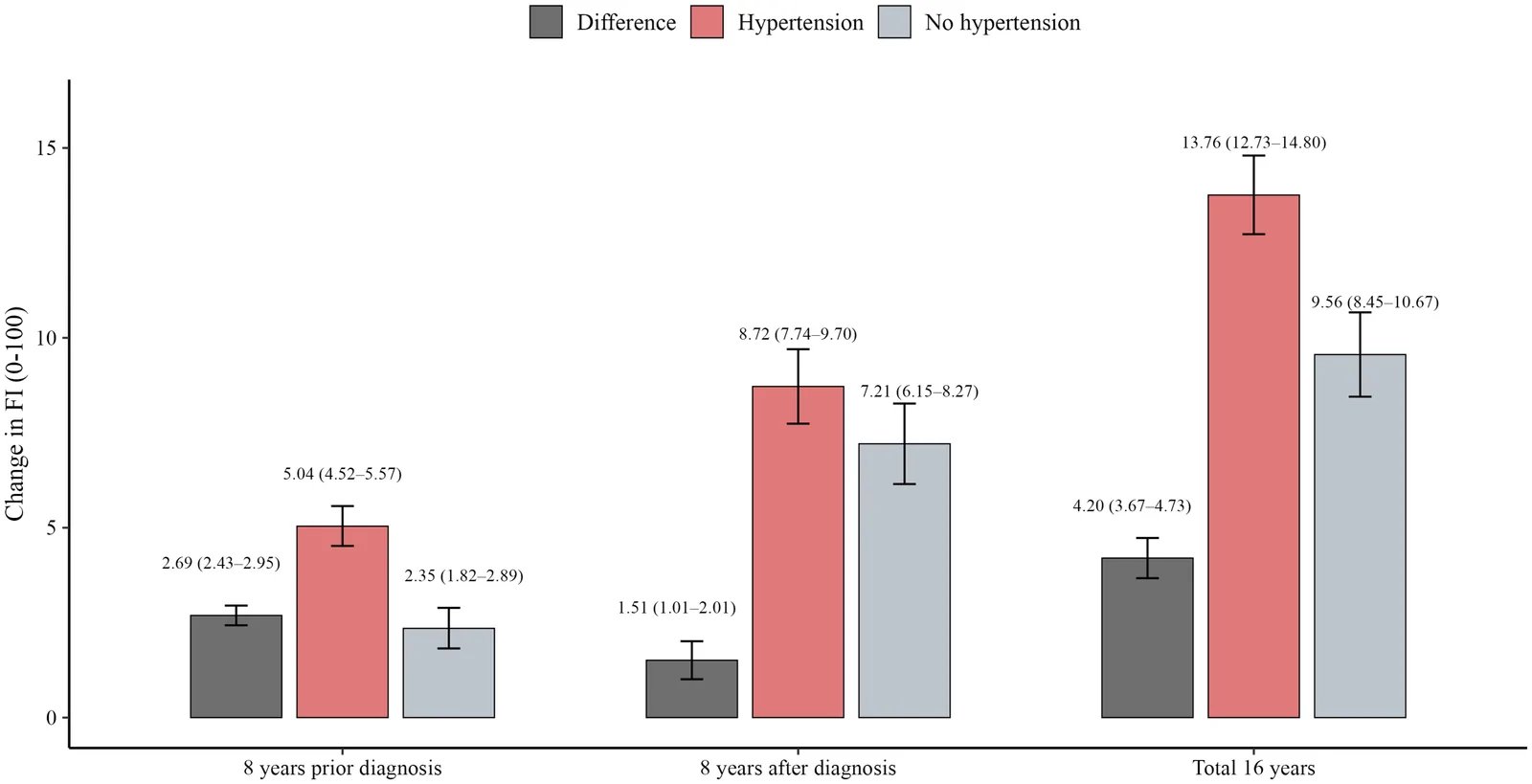
Changes in frailty index rescaled to a 0 to 100 scale (FI)_100_ before and after diagnosis of hypertension and differences across hypertension status.

The association of use of antihypertensive medication reported by participants with the rate of change in FI_100_ among individuals with hypertension after diagnosis (β=−0.05 [95% CI, −0.28 to 0.18]; *P*=0.653) was not significant. The association remained insignificant among frail individuals with hypertension (β=0.25 [95% CI, −0.48 to 0.99]; *P*=0.499). Figure 3 presents the frailty trajectories among individuals with hypertension with and without medication use.

**Figure 3.**
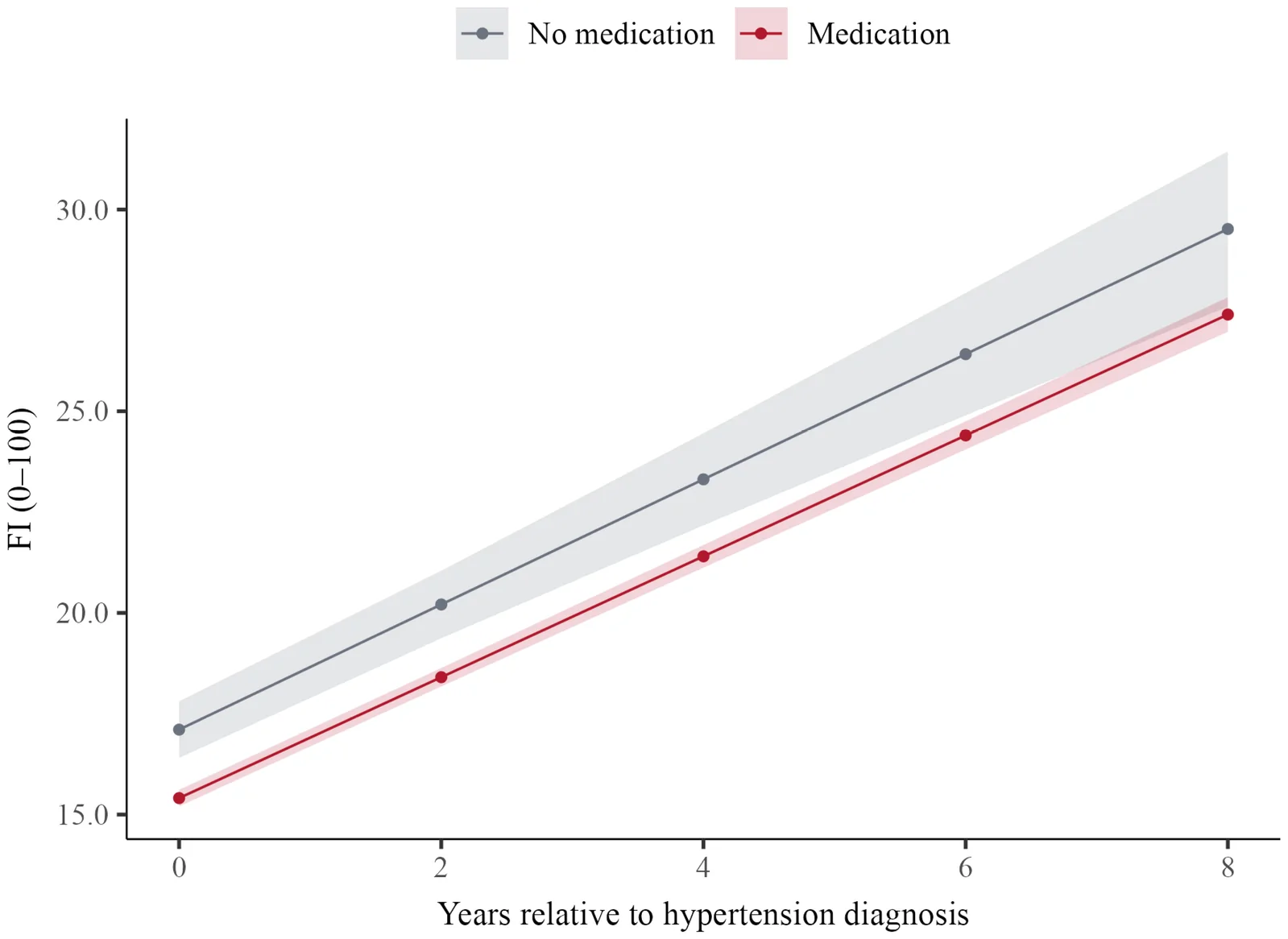
**Frailty trajectories across antihypertensive medication use status among individuals with hypertension after diagnosis.** Frailty index (FI) scores were rescaled to a 0 to 100 metric for analysis.

The positive interaction between hypertension, pretime, and age_t=0_ (β=0.03 [95% CI, 0.02–0.04]) suggests that among individuals with hypertension, older age of diagnosis was associated with a greater increase in FI_100_ per year in the years preceding diagnosis. The interaction between hypertension, posttime, and age_t=0_ was not significant (β=0.01 [95% CI, −0.01 to 0.03]). Leave-one-domain-out analyses are present in Table S4. Excluding any individual frailty domain produced only small changes in the prediagnosis and postdiagnosis slopes. The corresponding 95% CIs showed overlap across all models. Results were stable when analyses were repeated in the full sample (Table S5), the sample with at least 3 follow-ups (Table S6), the sample excluding frail individuals (FI ≥0.25) at baseline (Table S7), the sample excluding individuals with stroke or heart problems at baseline and follow-ups (Table S8), and the sample after adding household wealth quartiles as a matching variable (Table S9).

## Discussion

This study, using data from 3 representative cohorts covering 14 countries over up to 18 years of follow-up, found that participants with hypertension showed a faster increase in FI before and after diagnosis compared with those without a hypertension diagnosis. Among individuals with hypertension, antihypertensive medication was associated with slower progression of frailty, but this association was not statistically significant. Results remained robust in sensitivity analyses.

Previous cross-sectional and longitudinal studies have reported higher frailty prevalence or incidence among people with hypertension, although findings are inconsistent.^16^ Beyond hypertension status alone, accumulating evidence suggests that BP patterns over time may play an important role in frailty development.^18–20^ Poorly controlled BP and greater BP variability may contribute to progression of frailty.^18,19^ Evidence from Chinese populations suggests that persistently elevated or increasing systolic BP and higher cumulative BP are associated with incident frailty and increasing FI.^19,20^ Here, we extended these findings by characterizing frailty slopes before and after hypertension diagnosis among middle-aged and older adults across 14 countries and found that hypertension was associated with faster frailty accumulation.

During the 8 years before diagnosis, the hypertension group had a 0.03-point greater increase in FI (0–1 scale) than the nonhypertension group, equivalent to ≈0.7 additional health deficits captured by FI. Eight years after diagnosis, the hypertension group had accumulated a 0.02-point (0.4 deficits, 0–1 scale) greater increase in FI than the no hypertension group. Over 16 years, individuals with hypertension accumulated 0.14 FI points (3.7 health deficits, 0–1 scale), compared with 0.10 points (2.6 deficits, 0–1 scale) among those without hypertension. Overall, this corresponds to ≈1 additional health deficit among individuals with hypertension, representing a modest difference. Given the high prevalence of hypertension, with global estimates of 26.2% among individuals <50 years and 52.2% among those aged ≥50 years in high-income countries,^37^ this excess frailty accumulation may translate into a substantial burden of morbidity. Notably, the smaller postdiagnosis gap in frailty across hypertension status is consistent with evidence that variability in frailty trajectories narrows at older ages.^38^

It is well established that appropriate antihypertensive treatment reduces cardiovascular risk in people with hypertension.^39^ Large trials such as the HYVET (Hypertension in the Very Elderly Trial) and the SPRINT (Systolic Blood Pressure Intervention Trial) demonstrated cardiovascular benefits of antihypertensive treatment in older adults, and secondary analyses suggested these benefits were generally maintained across different frailty levels, although individuals with severe frailty were largely excluded.^40–43^ In addition, previous observational studies have reported mixed findings about the association of antihypertensive treatment with frailty. Some suggested higher frailty incidence among users of several antihypertensive drug classes, while others found no significant association between antihypertensive use and frailty risk.^29,28^ In our study, antihypertensive medication use was associated with a slower rate of frailty progression after hypertension diagnosis, but the association did not reach statistical significance. This may indicate that antihypertensive treatment does not substantially influence frailty trajectories, although modest protective effects cannot be excluded. However, interpretation of this finding should be cautious, as only a small proportion of participants with hypertension remained untreated (9.0%) during follow-up, which may have limited statistical power to detect meaningful differences. In addition, confounding by indication may also exist, as the decision to prescribe antihypertensive medication is influenced by factors like BP, comorbidity burden, and cardiovascular risk that potentially affect frailty progression. Moreover, the sensitivity analyses restricted to participants with frailty showed a nonsignificant association of medication with faster progression of frailty. This finding also needs cautious interpretation due to the potential for confounding by indication, competing risks of mortality, and the limited sample size in this subgroup. There is also the risk of immortal time bias in these types of analyses, as medication use could be reported at any wave from reported diagnosis. However, the majority of participants reported medication use at the wave of diagnosis, meaning the potential for this bias should be limited. In light of these potential biases, the nonsignificant association between medication and faster frailty progression may not suggest medication use is harmful in frailer populations, but highlights the complexity of treatment decisions in this group and the challenges of interpreting these associations in observational data.

The leave-one-out analyses demonstrated that exclusion of any single frailty domain did not materially alter the associations between hypertension and frailty progression. Although exclusion of the mobility domain resulted in a slightly greater attenuation of the prediagnosis slope, the 95% CI overlapped and overall estimates remained highly consistent across all analyses. These findings suggest that the accelerated frailty progression was not explained by any single component of the FI, but may reflect changes across multiple domains of frailty, including chronic conditions and physical health; activities of daily living and instrumental activities of daily living disabilities; mobility; and mental health and cognition. Some mechanisms may explain the differences in progression of frailty across hypertension status. Sustained vascular and physiological stress may contribute to accumulation of frailty long before hypertension is clinically recognized.^19,20^ Compared with those without hypertension, participants with hypertension might experience periods of undetected BP elevation, which is one of the major causes of clinical and preclinical damage to multiple organs and systems, thereby accelerating progression of frailty before diagnosis.^6,44^ However, because repeated BP measurements before diagnosis were unavailable, our study cannot distinguish this explanation from an alternative pathophysiological sequence. It is also possible that common biological mechanisms underlying both hypertension and frailty, like chronic inflammation, contribute to frailty accumulation first while also promoting the subsequent development of hypertension.^10,11^ In this scenario, the greater frailty accumulation among individuals with hypertension observed before diagnosis may result from biological processes that lead to both frailty and hypertension, rather than from subclinical hypertension alone. This supports the possibility that frailty and hypertension likely reinforce each other over time, rather than following a strictly unidirectional sequence. Both the hypertension group and the no hypertension group experienced an acceleration of frailty accumulation after diagnosis in this study. This probably reflects the nonlinear relationship between the FI and age around the mean age at diagnosis of 70 years.^45^ But the hypertension group still showed more rapid progression of frailty compared with the no-hypertension group after diagnosis. This may be explained by the higher burden of hypertension-related comorbidities which are associated with frailty, such as chronic kidney disease,^46^ or captured as health deficits in FI, such as cognitive impairment.^9,47^ In addition, hypertension-related impairments in cognition, mobility, and functional capacity may further contribute to accelerated accumulation of frailty,^6,48^ although the evidence is not uniform. Although some longitudinal studies have found that higher BP is associated with accelerated decline in gait speed, higher BP was found to be associated with better physical function among 85-year-old and older community-dwelling individuals.^49^ Moreover, the SPRINT trial found no effect of intensive BP lowering compared with standard treatment on gait speed or mobility outcomes among adults aged ≥75 years.^50^ These disparate findings suggest the relationship may be complex and affected by many factors, including age and duration and intensity of exposure and treatment. Of note, a smaller postdiagnosis gap in frailty progression between the hypertension group and no hypertension group was observed. This may partly reflect the convergence of frailty trajectories at older ages^51^ and could also partly be attributable to the initiation of antihypertensive treatment, although the effect did not reach statistical significance in our analysis.

These findings have multiple implications. Current guidelines strongly recommend screening for frailty in older adults.^52^ In parallel, hypertension guidelines emphasize periodic monitoring of BP and comprehensive baseline assessment at diagnosis to support cardiovascular risk stratification.^53,54^ Within this existing framework, assessment of frailty could be incorporated into routine hypertension care, particularly at the time of diagnosis and during follow-up. This could allow early identification of frailty and informed treatment decisions and BP management. Meanwhile, given that hypertension is often asymptomatic,^40^ individuals identified as frail should be considered for opportunistic BP screening during routine healthcare encounters. In addition, our study didn’t find the harms of initiating antihypertensive medication among adults with hypertension. Following recent trials including SPRINT and the ESPRIT trial (Effects of Intensive Systolic Blood Pressure Lowering Treatment in Reducing Risk of Vascular Events),^41,55^ the European Society of Cardiology and American Heart Association have already updated their guidelines and lowered the target BP for antihypertensive treatment to <130 mm Hg.^53,54^ Considering the early accumulation of deficits before meeting the hypertension diagnosis standard, reducing the threshold for hypertension treatment could be considered to postpone the progression of frailty. However, treatment in older adults should be individualized, given risks such as hypotension, falls, and polypharmacy.^11,56^ Integrating geriatric-informed, multidisciplinary care into cardiovascular services may optimize management for those with severe frailty.

Several limitations need to be noted. Selection and survival bias may exist and result in a relatively healthier and younger analytic sample. Individuals in poorer health may be more likely to be excluded or lost to follow-up, and the analytic sample excluded individuals with hypertension at baseline, who were, on average, older and had higher FI. As a result, the observed frailty trajectories may underestimate the true burden of frailty progression associated with hypertension. Accordingly, our findings may be more generalizable to individuals with later-onset hypertension rather than those with longstanding disease, although participants could have delayed diagnosis. Although multiple cohorts were pooled to increase sample size and generalizability, inherent differences between cohorts were combined, potentially introducing heterogeneity and measurement error. Hypertension was defined from self-reported doctor diagnosis rather than directly from health records or physiological measurements, and other key variables were self-reported. As a result, some individuals with undiagnosed hypertension may have been misclassified into the nonhypertension group, and the timing of diagnosis reflects clinical recognition rather than biological onset. Given that hypertension often develops asymptomatically, the true duration of exposure to hypertension before diagnosis is unknown and likely varies substantially across participants. This imprecision may attenuate the estimated differences in frailty progression across hypertension status. In addition, other key variables were self-reported, making them susceptible to recall bias. In addition, the possibility of detection bias should be considered: individuals with increasing frailty may have more frequent contact with healthcare services, leading to earlier diagnosis of hypertension. This might partly contribute to the observed elevation in FI among individuals diagnosed with hypertension in the prediagnosis period. Moreover, some key covariates were not consistently available across all 3 cohorts. For example, dietary patterns differ across countries, and the residual confounding could bias the association. Finally, participants aged <50 years were included in the analysis, and this population was not systematically sampled, which may introduce bias. But these limitations were well balanced by large samples drawn from representative cohort studies across 14 countries, long follow-up duration, and use of CEM to select a control group.

Future studies using electronic healthcare records and more objective longitudinal measurements of both frailty and hypertension could help to further clarify the association of hypertension and frailty. In particular, studies with more precise information on BP, treatment initiation, medication adherence, and treatment intensity could help distinguish the effects of hypertension itself from those confounders on progression of frailty. In addition, clinical trials may be warranted to explore the potential effects of different kinds of antihypertensive treatment on the progression of frailty.

In conclusion, participants with hypertension persistently experienced a faster progression of frailty both before and after clinical diagnosis compared with those without hypertension. Although the use of antihypertensive medications was associated with an attenuation of progression of frailty after diagnosis, the association did not reach statistical significance. These findings suggest the importance of incorporating assessment of frailty into the comprehensive risk evaluation at the time of hypertension diagnosis, as well as into subsequent follow-up care, alongside opportunistic BP screening in frail individuals. However, these findings warrant cautious interpretation and further studies using objective measurement of BP and precise timing of diagnosis are needed to confirm this association.

## Perspectives

Frailty progression appears to be already accelerated in the years preceding clinical hypertension diagnosis, and this acceleration continues after diagnosis. This temporal pattern suggests that the period around hypertension diagnosis represents an opportunity: incorporating frailty assessment into hypertension care could identify individuals at highest risk of rapid functional decline, allowing earlier targeting of supportive interventions. Conversely, an upward frailty trajectory in older adults may warrant closer attention to BP. Future work should use longitudinal and objective BP measurements and repeated frailty assessments to clarify the association of BP with frailty. In addition, randomized trials are needed to determine the effect of antihypertensive treatments on frailty.

## Article Information

### Author Contributions

D. Tang and M. O’Connell conceptualized this study. D. Tang analyzed the data. A. Goubar and M. O’Connell validated the data. D. Tang drafted the manuscript. A. Goubar, C. Pantoja-Ruiz, A. Bhalla, J. Whitney, M. Cai, W. Deng, S. Tang, I.J. Marshall, C.D.A. Wolfe, and M. O’Connell reviewed and revised the manuscript. M. O’Connell supervised this study. All authors reviewed and approved the final manuscript.

### Disclosures

None.

## Supplementary Material


