# Supplementary material for: Frailty Index Progression Around Hypertension Diagnosis in 14 Countries
Source: Hypertension. 2026 Sep 30;83(11):e27590. doi: 10.1161/HYPERTENSIONAHA.126.27590 (PMC13680766; doi:10.1161/HYPERTENSIONAHA.126.27590)
Supplement: Supplementary file 1 [file hyp-83-e27590-s001.pdf]

Supplementary materials

Frailty Index Progression Around Hypertension Diagnosis in 14 Countries

Dongfeng Tang MSc [a], Aicha Goubar PhD [a], Camila Pantoja-Ruiz MSc [a], Ajay Bhalla MD [b], Julie Whitney PhD [a, c], Miao Cai PhD [d], Wuquan Deng MD [e], Shangfeng Tang PhD [f,g], Iain J. Marshall MRCGP [a], Charles D A Wolfe MD [a,h], Matthew DL O'Connell PhD [a]

[a]: School of Life Course and Population Sciences, Faculty of Life Science and Medicine, King's College London, London, UK
[b]: Guys and St Thomas NHS Foundation Trust and School of Life Course and Population Sciences, King's College London, London, UK
[c]: Department of Clinical Gerontology, King's College Hospital NHS Foundation Trust, London, UK
[d]: Clinical Epidemiology Center, Research and DevelopmenFt Service, VA Saint Louis Health Care System, Saint Louis, MO, USA
[e]: School of Medicine, Department of Endocrinology, Chongqing University Central Hospital, Chongqing University, Chongqing, China
[f]: School of Medicine and Health Management, Huazhong University of Science and Technology, Hubei, China
[g]: Research Center for Rural Health Service, Key Research Institute of Humanities & Social Sciences of Hubei Provincial Department of Education, Hubei, China
[h]: NIHR Applied Research Collaboration (ARC) South London, London, United Kingdom

Corresponding authors
Dongfeng Tang
School of Life Course and Population Sciences,
Faculty of Life Science and Medicine,
King's College London, London, UK

Guy’s campus, Great Maze Pond
London, SE1 1UL
UK
Email: dongfeng.tang@kcl.ac.uk
Phone: 44+7522762135

Shangfeng Tang
School of Medicine and Health Management,
Huazhong University of Science and Technology,
Hubei, China

No. 13, Aviation Road, Qiaokou District
Wuhan, 430030
China
Email: sftang2018@hust.edu.cn
Phone: 86+13349895639

Table of Contents

Supplementary methods 3

Table S1 Included frailty index items 4

Table S2 Characteristics of excluded baseline hypertensive participants and remained hypertensive participants 6

Table S3 Classification of FI items into four domains 7

Table S4 Leave-one-domain-out sensitivity analysis 8

Table S5 Sensitivity analyses using sample with matched and not matched participants 9

Table S6 Sensitivity analyses using the sample with at least three follow-ups 10

Table S7 Sensitivity analyses using the sample after excluding frail participants in baseline 11

Table S8 Sensitivity analyses using the sample after excluding participants with stroke and heart problems at baseline 12

Figure S1 Flowchart of participants' inclusion and exclusion 14

Figure S2 SMDs of FI and covariates before and after matching with total wealth added as a matching variable 15

Supplementary methods

Measurements

Age in the investigated year and gender were self-reported. Marital status was grouped into partnered (married and living together with/ separated from spouse; partnered) and single (Never married; Divorced; Widowed). Education level was harmonized based on a simplified version of 1997 International Standard Classification of Education (ISCED-97) codes and groped into three levels: primary, secondary, and tertiary. Total household wealth was self-reported and calculated first by summing all wealth components (residence, business, vehicles, and savings accounts), excluding debts or loans [1]. Then, total wealth quartiles were included into analysis. Smoking and drinking were dichotomized as yes or no according to whether ever smoked or drank alcohol. Physical inactivity was assessed by activity types and frequencies. Participants taking part in vigorous/moderate physical activity more than once a week or once a week were coded as no [2]. Otherwise, they were grouped as yes. Country was included as a categorial variable including 14 countries: England, America, Austria, Germany, Sweden, Netherlands, Spain, Italy, France, Denmark, Greece, Switzerland, Belgium, and Israel. Birth cohort was created based on birth year and grouped every five years from 1900 to 1982. Birth cohort was included as a continuous variable centred on the 1940–1944 cohort to improve model parsimony.

Matching

CEM package in Stata was used to carry out coarsened exact matching, using the k-to-k procedure to create hypertension groups with equal number of participants. Age, marital status and FI at baseline, gender, education, birth cohort, and country were chosen as the matching variables mainly on the consideration of assuring baseline balance while minimizing data loss. Other factors like smoking, household wealth, smoking, drinking, and physical inactivity were adjusted in the model rather than using them as matching variables.

To simplify analyses and reporting, a *k*-to-*k* matching procedure was employed. Within the CEM framework, valid causal inference requires that equal numbers of treated and control units be maintained within each matched stratum. Although all matched observations can be retained by applying CEM-derived weights to adjust for unequal group sizes across strata, the inclusion of additional covariates beyond the matching variables renders the appropriate weighting strategy unclear. These complications are avoided by the *k*-to-*k* procedure, which preserves within-stratum balance and allows valid causal inference without the need for model weighting.

Table S1 Included frailty index items

No	Description of the item			Cut-off value
	ELSA	HRS	SHARE	
1	Self-reported physician diagnosed diabetes			Yes = 1, No = 0
2	Self-reported physician diagnosed cancer			Yes = 1, No = 0
3	Self-reported physician diagnosed arthritis			Yes = 1, No = 0
4	Self-reported physician diagnosed chronic lung disease			Yes = 1, No = 0
5	Self-reported diagnosed memory-related disease, including Alzheimer's disease or dementia, organic brain senility, or other serious memory impairment			Yes = 1, No = 0
6[a]	Self-reported physician diagnosed any emotional, nervous, or psychiatric problems			Yes = 1, No = 0
7[b]	CESD-8		EURO-D	CESD-8 or EURO-D ≥4: 1<br>CESD-8 or EURO-D <4: 0
8	Self-reported general eyesight		Self-reported eyesight for seeing thing up close	Blind, poor or fair=1, Good,<br>very good or excellent=0
9	Self-reported hearing while wearing hearing aid or as usual			Deaf, poor or fair=1, Good,<br>very good or excellent=0
10	Self-reported general health status			Very poor or poor = 1, Very good, good, or fair = 0
11	Difficulty with dressing			Yes = 1, No = 0
12	Difficulty with bathing or showering			Yes = 1, No = 0
13	Difficulty with eating			Yes = 1, No = 0
14	Difficulty with getting in and out of bed			Yes = 1, No = 0
15	Difficulty with using the toilet			Yes = 1, No = 0
16	Difficulty with managing money			Yes = 1, No = 0
17	Difficulty with taking medication			Yes = 1, No = 0

18	Difficulty with shopping for groceries	Yes = 1, No = 0
19	Difficulty with preparing meals	Yes = 1, No = 0
20	Mobility: difficulty with walking 100 yards or one block	Yes = 1, No = 0
21	Mobility: difficulty with getting up from a chair after sitting for long periods	Yes = 1, No = 0
22	Mobility: difficulty with climbing several flights of stairs without resting	Yes = 1, No = 0
23	Mobility: difficulty with lifting or carrying weights over 10 pounds/jins	Yes = 1, No = 0
24	Mobility: difficulty with picking up a coin from the table	Yes = 1, No = 0
25	Mobility: difficulty with stooping, kneeling, or crouching	Yes = 1, No = 0
26	Mobility: difficulty with reaching arms above shoulder level	Yes = 1, No = 0
27[c]	Cognition: (total score - (immediate and delayed word recall + date naming)) / total score	Continuous variable, ranging from 0 to 1

[a]: It should be noted that there was no diagnosis data of emotional, nervous or psychiatric problems in the data of wave 1 and 3 from SHARE. Therefore, this item was not included when calculating FI in wave 1 and wave 3 in SHARE.

[b]: In HRS and ELSA, the 9-item Center for Epidemiologic Studies Depression Scale (CESD-8) with a total score ranging from zero to eight was used to assess depression and participants with a score of four or over were defined as having depression. The 12-item EURO-D was used in SHARE. It ranges from zero to 12, with a summary score ≥4 indicating depression.

[c]: Cognition test consists of three components, including immediate, delayed word recall, and date naming. For word recall, participants are required to recite 10 words. For date naming, participants are asked whether they could remember the date of that day (day ofweek, day of month, month, and year. One score is given for each right answer, and the cognition score is calculated by the formula: (total score-(immediate and delayed word recall + date naming))/total score. Total score refers to the theoretical maximum score of all tests. Hence, a higher cognition score indicated lower cognition function. Cognition score in wave 16 of HRS was not available, therefore it was not included when calculating FI in wave 16 in HRS.

Table S2 Characteristics of excluded baseline hypertensive participants and remained hypertensive participants

		Remained Hypertensive participants	Excluded baseline hypertensive participants	p-value
		N=10,709	N=11,715	
FI (Mean, SD)		0.13 (0.12)	0.19 (0.15)	<0.001
Age (Mean, SD)		63.1 (9.5)	67.0 (9.1)	<0.001
Gender				0.035
	Male	4,771 (44.6%)	5,055 (43.1%)	
	Female	5,938 (55.4%)	6,660 (56.9%)	
Household wealth (quartile)				0.23
	Q1	3,066 (28.6%)	3,450 (29.4%)	
	Q2	3,037 (28.4%)	3,330 (28.4%)	
	Q3	2,684 (25.1%)	2,805 (23.9%)	
	Q4	1,922 (17.9%)	2,130 (18.2%)	
	Education			<0.001
	Less than upper secondary	4,286 (40.0%)	6,001 (51.2%)	
	Upper secondary and vocational training	4,310 (40.2%)	3,890 (33.2%)	
	Tertiary	2,113 (19.7%)	1,824 (15.6%)	
Drinking				<0.001
	No	3,355 (31.3%)	4,736 (40.4%)	
	Yes	7,354 (68.7%)	6,979 (59.6%)	
Smoking				<0.001
	No	5,046 (47.1%)	6,140 (52.4%)	
	Yes	5,663 (52.9%)	5,575 (47.6%)	
	Physical inactivity			<0.001
	No	8,961 (83.7%)	9,138 (78.0%)	
	Yes	1,748 (16.3%)	2,577 (22.0%)	

Table S3 Classification of FI items into four domains

Domain	Items
Domain 1: Chronic conditions and physical health (7 items)	Self-reported physician diagnosed diabetes
	Self-reported physician diagnosed cancer
	Self-reported physician diagnosed arthritis
	Self-reported physician diagnosed chronic lung disease
	Eyesight (self-reported eyesight)
	Hearing (self-reported hearing)
	Self-rated general health
Domain 2: ADL and IADL disabilities (9 items)	Difficulty with dressing
	Difficulty with bathing or showering
	Difficulty with eating
	Difficulty with getting in and out of bed
	Difficulty with using the toilet
	Difficulty with managing money
	Difficulty with taking medication
	Difficulty with shopping for groceries
	Difficulty with preparing meals
Domain 3: Mobility (7 items)	Mobility: difficulty with walking 100 yards or one block
	Mobility: difficulty with getting up from a chair after sitting for long periods
	Mobility: difficulty with climbing several flights of stairs without resting
	Mobility: difficulty with lifting or carrying weights over 10 pounds/jins
	Mobility: difficulty with picking up a coin from the table
	Mobility: difficulty with stooping, kneeling, or crouching
	Mobility: difficulty with reaching arms above shoulder level
Domain 4: Mental health & cognition (4 items)	Depressive symptoms (CESD-8 / EURO-D)
	Cognition: (total score - (immediate and delayed word recall + date naming)) / total score
	Self-reported diagnosed memory-related disease, including Alzheimer's disease or dementia, organic brain senility, or other serious memory impairment
	Self-reported physician diagnosed any emotional, nervous, or psychiatric problems

Table S4 Leave-one-domain-out sensitivity analysis

FI	Pre-diagnosis slope difference/year (95% CI)	Post-diagnosis slope difference/year (95% CI)
Main analysis	0.34 (0.30, 0.37)	0.19 (0.13, 0.25)
Excluding chronic conditions and physical health	0.37 (0.33, 0.40)	0.20 (0.12, 0.28)
Excluding ADL and IADL disabilities	0.34 (0.30, 0.37)	0.16 (0.11, 0.22)
Excluding mobility	0.29 (0.26, 0.31)	0.17 (0.11, 0.23)
Excluding mental health &cognition domain	0.35 (0.31, 0.38)	0.21 (0.14, 0.28)

Table S5 Sensitivity analyses using sample with matched and not matched participants

	coefficient	P-value	95% CI	
			Lower	Upper
**At baseline**				
No hypertension	14.72	P<0.001	14.08	15.37
Hypertension	19.16	P<0.001	18.51	19.81
Hypertension-no hypertension	4.44	P<0.001	4.14	4.74
**Slope before diagnosis/median diagnosis**				
No hypertension	0.31	P<0.001	0.25	0.36
Hypertension	0.65	P<0.001	0.59	0.70
Hypertension-no hypertension	0.34	P<0.001	0.31	0.37
**Slope after diagnosis/median diagnosis**				
No hypertension	0.90	P<0.001	0.79	1.01
Hypertension	1.07	P<0.001	0.97	1.17
Hypertension-no hypertension	0.17	P<0.001	0.12	0.23

Table S6 Sensitivity analyses using the sample with at least three follow-ups

	coefficient	P-value	95% CI	
			Lower	Upper
**At baseline**				
No hypertension	15.41	P<0.001	14.39	16.43
Hypertension	18.73	P<0.001	17.78	19.68
Hypertension-no hypertension	3.32	P<0.001	2.86	3.79
**Slope before diagnosis/median diagnosis**				
No hypertension	0.28	P<0.001	0.16	0.40
Hypertension	0.63	P<0.001	0.51	0.75
Hypertension-no hypertension	0.35	P<0.001	0.29	0.41
**Slope after diagnosis/median diagnosis**				
No hypertension	0.87	P<0.001	0.73	1.01
Hypertension	1.00	P<0.001	0.88	1.13
Hypertension-no hypertension	0.13	P<0.001	0.07	0.20

Table S7 Sensitivity analyses using the sample after excluding frail participants in baseline

	coefficient	P-value	95% CI	
			Lower	Upper
**At baseline**				
No hypertension	10.86	P<0.001	10.19	11.52
Hypertension	15.05	P<0.001	14.40	15.70
Hypertension-no hypertension	4.20	P<0.001	3.90	4.49
**Slope before diagnosis/median diagnosis**				
No hypertension	0.33	P<0.001	0.27	0.39
Hypertension	0.66	P<0.001	0.61	0.72
Hypertension-no hypertension	0.33	P<0.001	0.30	0.36
**Slope after diagnosis/median diagnosis**				
No hypertension	0.95	P<0.001	0.81	1.10
Hypertension	1.20	P<0.001	1.06	1.33
Hypertension-no hypertension	0.24	P<0.001	0.18	0.31

Table S8 Sensitivity analyses using the sample after excluding participants with stroke and heart problems at baseline

	coefficient	P-value	95% CI	
			Lower	Upper
**At baseline**				
No hypertension	13.12	P<0.001	12.28	13.97
Hypertension	16.20	P<0.001	15.37	17.05
Hypertension-no hypertension	3.09	P<0.001	2.71	3.46
**Slope before diagnosis/median diagnosis**				
No hypertension	0.23	P<0.001	0.17	0.30
Hypertension	0.50	P<0.001	0.43	0.56
Hypertension-no hypertension	0.26	P<0.001	0.23	0.30
**Slope after diagnosis/median diagnosis**				
No hypertension	0.75	P<0.001	0.60	0.90
Hypertension	0.88	P<0.001	0.74	1.02
Hypertension-no hypertension	0.13	P<0.001	0.06	0.21

Table S9 Sensitivity analyses using the sample after adding total wealth as a matching variable

	coefficient	P-value	95% CI	
			Lower	Upper
**At baseline**				
No hypertension	14.50	P<0.001	13.73	15.27
Hypertension	18.85	P<0.001	18.09	19.61
Hypertension-no hypertension	4.35	P<0.001	4.00	4.69
**Slope before diagnosis/median diagnosis**				
No hypertension	0.30	P<0.001	0.23	0.37
Hypertension	0.63	P<0.001	0.57	0.70
Hypertension-no hypertension	0.34	P<0.001	0.30	0.37
**slope after diagnosis/median diagnosis**				
No hypertension	0.93	P<0.001	0.80	1.06
Hypertension	1.10	P<0.001	0.98	1.23
Hypertension-no hypertension	0.17	P<0.001	0.11	0.24

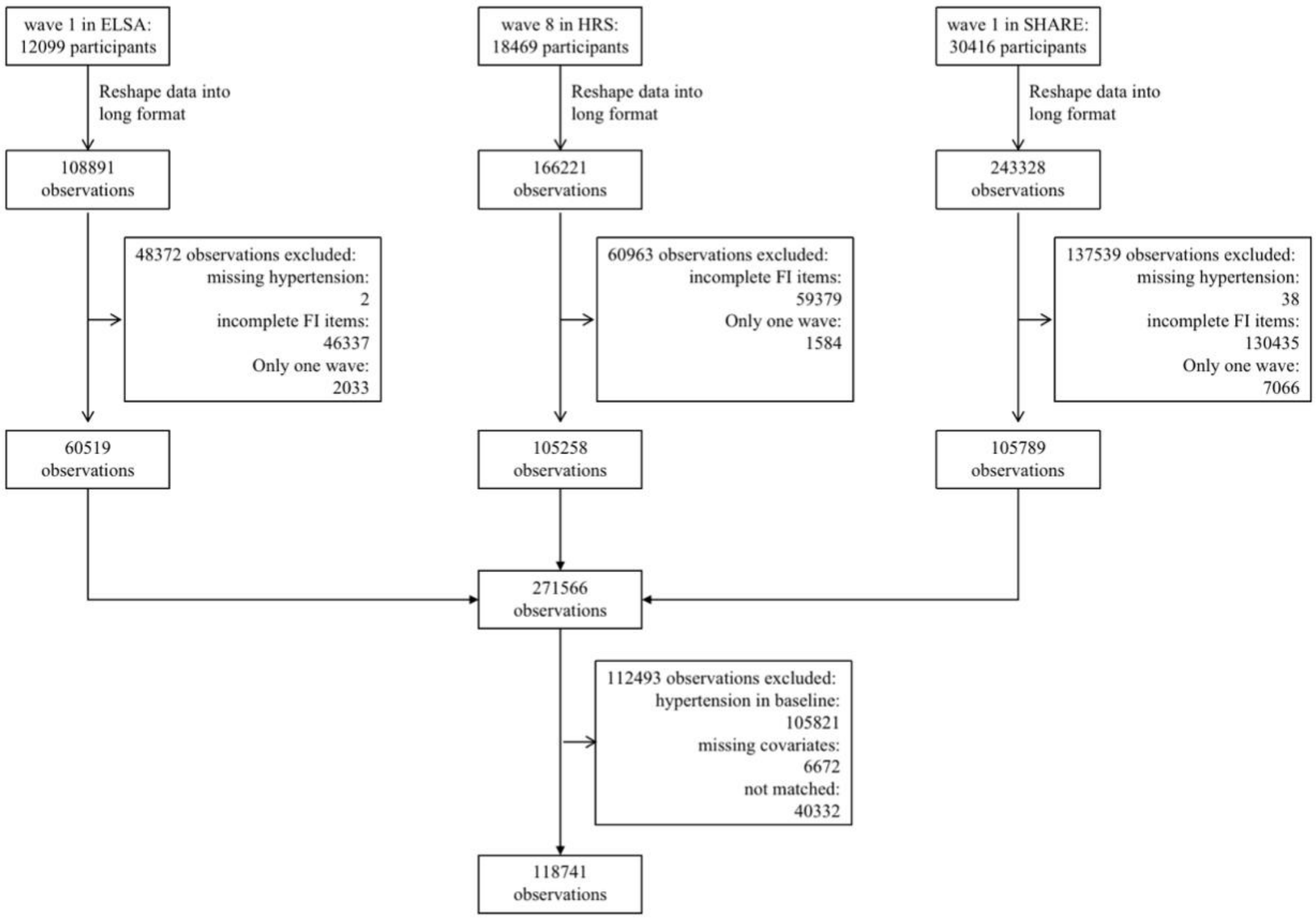

Figure S1 Flowchart of participants' inclusion and exclusion

Figure S2 SMDs of FI and covariates before and after matching with total wealth added as a matching variable

Reference:

1. Zhou Y, Kivimäki M, Yan LL, Carrillo-Larco RM, Zhang Y, Cheng Y, et al. Associations between socioeconomic inequalities and progression to psychological and cognitive multimorbidities after onset of a physical condition: A multicohort study. *EClinicalMedicine*. 2024;74:102739
2. Wang Y, Liu M, Yang F, Chen H, Wang Y, Liu J. The associations of socioeconomic status, social activities, and loneliness with depressive symptoms in adults aged 50 years and older across 24 countries: Findings from five prospective cohort studies. *Lancet Healthy Longev*. 2024;5:100618
